# Empiric antimicrobial therapy in the intensive care unit based on the risk of multidrug-resistant bacterial infection: a single-centre case‒control study of blood culture results in Japan

**DOI:** 10.1186/s13756-023-01303-2

**Published:** 2023-09-12

**Authors:** Taikan Nanao, Hideo Nishizawa, Junichi Fujimoto

**Affiliations:** 1 Department of Intensive Care Medicine, Yokohama Rosai Hospital, 3211, Kozukue, Kouhoku, Yokohama, Kanagawa 222-0036 Japan; 2 Graduate School of Medicine, International University of Health and Welfare, Tokyo, Japan

**Keywords:** Blood culture, *Pseudomonas aeruginosa*, Bacteraemia, Sepsis, Multidrug-resistant bacteria, Empiric therapy

## Abstract

**Background:**

Infections and sepsis are the leading causes of death in intensive care units (ICUs). Antimicrobial agent selection is challenging because the intervention is directly related to the outcome, and the problem of antimicrobial resistance (AMR) must be considered. Therefore, in this study, we aimed to clarify the epidemiological data and examine whether the detection rate of multidrug-resistant (MDR) bacteria differed depending on the presence or absence of the risk of MDR bacterial infections to establish guidance regarding the choice of antimicrobial therapy for ICU patients.

**Methods:**

This retrospective case‒control study was performed in a single ICU in Japan. Patients admitted to the ICU who underwent blood culture (BC) analysis were considered for inclusion in this study; patients were at risk of MDR bacterial infections, and controls were not. The primary outcome measure was the detection rate of MDR bacteria in BCs collected from patients and controls. The secondary outcome measure was the selection rate of anti-*Pseudomonas* and anti-methicillin-resistant *Staphylococcus aureus* (MRSA) drugs for patients and controls.

**Results:**

Among the 1,730 patients admitted to the ICU during the study period, BCs were obtained from 186 patients, and 173 samples were finally included in the analysis (n = 129 cases; n = 44 controls). No MDR bacteria or *Pseudomonas aeruginosa* were detected in the controls (14 (11%) vs. 0 (0%)) (*P* = 0.014) However, there was no difference in empiric antimicrobials, including anti-MRSA (30 (23%) vs. 12 (27%)) (*P* = 0.592) and anti-*Pseudomonas aeruginosa* (61 (47%) vs. 16 (36%)) (*P* = 0.208) drugs, that were administered to the two groups.

**Conclusions:**

Even in critically ill patients in the ICU, MDR bacteria are unlikely to be detected in patients without the risk of MDR bacterial infections. Therefore, for such patients, a strategy of starting empiric narrow-spectrum antimicrobial therapy rather than empiric broad-spectrum therapy should be considered. This strategy, in conjunction with daily updates of clinical and epidemiological data at each facility, will promote the appropriate use of antimicrobials and reduce the emergence of MDR bacteria in the ICU.

*Trial registration*: None.

## Background

Although intensive care unit (ICU) beds in many hospitals account for less than 10% of all hospital beds, more than 20% of all hospital-acquired infections (HAIs) occur in the ICU [1]. Infections and sepsis are the leading causes of death and account for 40% of health care costs in ICUs [2]. Thus, ICU patients with organ damage for a variety of reasons are often considered to have sepsis or septic shock and are given a broad-spectrum antimicrobial agent. The Extended Study on Prevalence of Infection in Intensive Care III, an international multicentre observational study, showed that 54% of ICU patients had infectious diseases and that 70% of patients received antimicrobials [3]. In addition, the EUROBACT 1 study, another international multicentre observational study, showed that in 48% of ICU patients, bacteraemia was caused by multidrug resistant (MDR) bacteria [4].

In the context of this high infection burden among ICU patients, effective antimicrobial agent selection has become challenging not only because the intervention is directly related to the outcome [5, 6] but also because the problem of antimicrobial resistance (AMR) must be considered [7], which is becoming increasingly serious worldwide [8]. In addition, the risk of in-hospital mortality, renal impairment, and *Clostridioides difficile* infection (CDI) is significantly higher among patients given empiric multidrug-resistant (MDR) bacterium-targeting antimicrobials with undetectable MDR organisms than among those with detectable MDR organisms [9]. Therefore, the use of not only antimicrobials with inadequate activity spectra but also those with unnecessarily broad-spectrum activity should be avoided.

Specifically, when selecting empiric antimicrobial agents, we must decide whether to include treatments targeting *Pseudomonas aeruginosa* and MDR bacteria such as methicillin-resistant *Staphylococcus aureus* (MRSA) and bacteria that produce extended-spectrum β-lactamase (ESBL). The guidelines of the Surviving Sepsis Campaign: International Guidelines for Management of Sepsis and Septic Shock 2021 (SSCG2021) and the Japanese Clinical Practice Guidelines for Management of Sepsis and Septic Shock 2020 (J-SSCG 2020) indicate when MDR bacteria should be included as targets for antimicrobial treatment [10, 11]. However, the level of evidence on which this guidance is based is very low due to clinical heterogeneity, including in terms of patient characteristics, infection sources, causative agents, and antibiotic resistance patterns [10].

In Japan, an antimicrobial resistance action plan was adopted in 2015. However, the isolation rate of resistant bacteria has not decreased, and the 2020 targets were not reached for MRSA (47.7% in 2019 vs. the target value of 20% or lower) or carbapenem-resistant *Pseudomonas aeruginosa* (10.6% in 2019 vs. the target value of 10% or lower). Therefore, we also need to use broad-spectrum antimicrobial agents in specific cases in which MDR bacteria are likely to be the causative agents. When a critically ill patient in the ICU is suspected of having a concurrent infection, broad-spectrum antimicrobial agents tend to be used after blood culture (BC) testing is performed, as the failure of empiric therapy is likely to worsen the patient's prognosis. Essentially, the choice of broad-spectrum antimicrobial agents should be based primarily on the risk of MDR bacterial infection, not on the severity of illness, because antimicrobials are designed to kill the bacteria. However, there is no clear indicator of when a broad-spectrum antimicrobial should be administered in cases of suspected infection in the ICU for which BCs were obtained.

Appropriate empiric antimicrobials vary from institution to institution, and the appropriate treatment is not provided in the guidelines. Therefore, it is essential to understand the clinical and epidemiological data obtained from BCs at each facility to achieve the appropriate use of empirical antimicrobials. To establish guidance regarding the choice of antimicrobial therapy for ICU patients, we clarified the clinical characteristics, bacteriological test results, treatments, and prognoses of all patients with BCs collected in the ICU and examined whether the detection rate of MDR bacteria differed depending on the presence or absence of the risk of MDR bacterial infections.

## Methods

### Definitions

The following definitions were used:

#### MDR bacteria

Bacteria that produce ESBL or carbapenemase, methicillin-resistant *Staphylococcus aureus* (MRSA), vancomycin-resistant *Enterococcus spp.*, or a pathogen resistant to three or more antimicrobial classes were defined as MDR bacteria.

#### Broad-spectrum antimicrobials

Antimicrobials classified as “watch” or “reserve” in the World Health Organization Essential Medicines List Antibiotic Book [12] and with anti-*Pseudomonas spp.* or anti-MRSA activity were classified as broad-spectrum antimicrobials. Other antimicrobials were defined as narrow-spectrum antimicrobials in this study.

### Participants

This was a retrospective observational case‒control study conducted in a mixed emergency/medical/surgical ICU with 10 beds at Yokohama Rosai Hospital, Kanagawa, Japan, which has a 650-bed capacity and at which organ transplants are not performed. Patients admitted to the ICU between September 2019 and December 2021 who underwent BC analysis were considered for inclusion. In our ICU, infection is suspected when new organ damage is observed, lactate levels rise, or there are fluctuations in vital signs or an increased inflammatory response that cannot be explained by other factors. Then, physicians in the ICU decide to order two or more BC tests to increase the detection rate of bacteria and to facilitate the determination of contamination when infection is suspected. The first BCs performed during the first ICU admission during a single hospitalization were included in the analysis. A BC from the same patient was included in the analysis if he or she was discharged and then readmitted to the ICU. Then, we excluded cases that met any of the following three criteria: those with BCs performed to confirm other negative BC results, those with BCs routinely taken prior to prophylactic antimicrobial administration during targeted temperature management (TTM) therapy following return of spontaneous circulation after cardiac arrest to a precise target temperature between 34 and 37.5 °C for 72 h and those of patients under 18 years of age.

### Measurements

#### General clinical data

We collected data regarding age, sex, body mass index (BMI), coexisting conditions (chronic obstructive pulmonary disease (COPD), diabetes, dialysis, malignancy, allergy to antibiotics), implantable devices, admission source, Sequential Organ Failure Assessment (SOFA) score, ΔSOFA score (the SOFA score on the day of BC collection minus the SOFA score on the previous day), and lactate level at the time of BC collection from the medical record. For coexisting conditions, we extracted data that are generally used as a reference for empirical antimicrobial selection. For the SOFA score, we extracted data once daily during the period of ICU admission. We also collected information on infection site, culture results from blood, empiric antimicrobials, whether de-escalation was implemented or not, length of ICU stay, length of hospital stay, number of ventilator-free days (VFDs), 28-day mortality, discharge route, appropriate antimicrobial spectrum coverage rate for BC-positive cases and CDI and candidemia after ICU discharge. Finally, we also collected information on treatment in the ICU: intubation, tracheostomy, thoracic drainage, invasive positive pressure ventilation (IPPV), noninvasive positive pressure ventilation (NPPV), high-flow oxygen therapy (HFOT), central venous line (CVL) insertion, peripherally inserted central venous catheter (PICC) insertion, renal replacement therapy (RRT), intra-aortic balloon pumping (IABP), extracorporeal membrane oxygenation (ECMO), TTM, enteral nutrition (EN), and total parenteral nutrition (TPN).

#### Diagnoses

In our hospital, sepsis and septic shock are diagnosed according to the guidelines of the Third International Consensus Definitions for Sepsis and Septic Shock (Sepsis-3) as follows [13]: a diagnosis of sepsis was confirmed when the SOFA score acutely increased by 2 points or more in the presence of a clear infection or suspected infection. Patients with septic shock can be identified based on clinical manifestations of sepsis with persistent hypotension requiring vasopressors to maintain the mean blood pressure ≧ 65 mmHg and a serum lactate level > 2 mmol/L (18 mg/dL) despite adequate volume resuscitation.

#### BC data

Data on the infection site, whether the patient had bacteraemia, and whether empirical antimicrobials were administered were also collected. We defined empiric antibiotics as the first antibiotic administered after BC collection. The percentages of cases of bacteraemia were categorized based on patient characteristics (septic shock, sepsis without shock, and no sepsis) at the time of BC collection. Finally, we collected information on bacteria detected in BCs and antimicrobial agents selected as empirical antimicrobial agents. We collected at least two sets of BCs percutaneously. Then, we performed bacterial identification and drug susceptibility testing using Vitec 2 (Biomérieux, Japan). All bacteria detected in BCs were considered causative organisms with the exception of *Staphylococcus epidermidis*. If *Staphylococcus epidermidis* was detected in two or more sets of BCs, it was determined to be the causative organism, but if it was detected in only one set, it was determined clinically to be the causative organism. The primary objective was to examine whether the detection rate of MDR bacteria differed depending on the presence or absence of the risk of MDR bacterial infections. The secondary outcome measure was the selection rate of anti-*Pseudomonas* and anti-methicillin-resistant *Staphylococcus aureus* (MRSA) drugs for patients and controls.

### Study group definitions

The included patients were divided into two groups according to the presence or absence of the risk of MDR bacterial infections. To assess the risk of MDR bacterial infections, we investigated the most relevant factors: history of detection of resistant organisms (A), history of antimicrobial use within 90 days (B), and history of hospitalization and institutionalization within 90 days (C) [11]. We selected two major factors commonly identified in many studies as risk factors for MDR bacterial infections: history of detection of MDR bacteria and history of antimicrobial therapy [14–16]. Then, we added a history of hospitalization and institutionalization, which is a risk factor for *Pseudomonas aeruginosa* infection [17], as a third risk factor for MDR bacterial infections because the decision to use an anti-*Pseudomonas aeruginosa* agent is very important during the selection of an empirical antimicrobial agent. *Pseudomonas* *aeruginosa* is a frequent cause of health care-associated bacteraemia [18] and one of the three most frequently isolated pathogens in patients with catheter-associated urinary tract infection and ventilator-associated pneumonia [19]. Those who met any of the risks (A-C) above were assigned to the R group, and those who did not were assigned to the control group.

### Statistical analysis

The percentages of cases of bacteraemia were categorized based on patient characteristics (septic shock, sepsis without shock, and no sepsis) at the time of BC collection. Differences between the R group with a risk of MDR bacterial infection and the control group without a risk of MDR bacterial infection were assessed using chi-square tests or Fisher’s exact tests for categorical variables and Mann–Whitney U tests for continuous variables. Differences with a two-tailed *P* value < 0.05 were considered statistically significant. All statistical analyses were performed using SPSS (version 26; IBM-SPSS Inc., Armonk, NY).

### Ethical considerations

Institutional approval was obtained from the Yokohama Rosai Hospital Ethics Committee (approval number 2021-18). Informed consent was obtained in an opt-out format.

## Results

### Patients

**Fig. 1:**
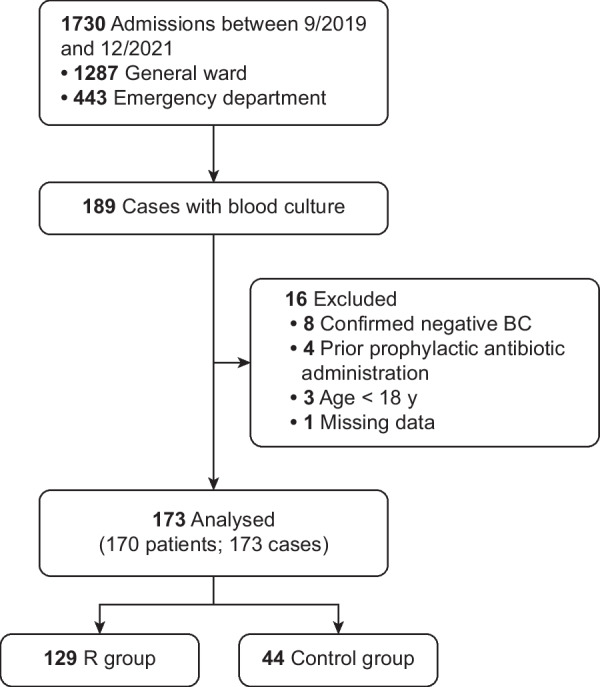
Study flow chart. The sample sizes are shown in bold text

During the study period, 1,730 patients were admitted to the ICU, among whom 189 underwent BC testing. We excluded a total of 16 cases; 15 were excluded according to the exclusion criteria, and one case had missing data. Thus, 173 cases among 170 patients were included in the analysis, as shown in Fig. 1.

The clinicodemographic characteristics of the study population for each group at the time of the BC test are shown in Table 1. The average age was 73 years, and 69 participants (40%) were women. Of the 173 cases with a BC, 129 (74.6%) regarded patients who exhibited at least one of our defined risks of infection with MDR bacteria, and 44 (25.4%) regarded patients who did not; these patients were assigned to the R and control groups, respectively. There were more patients with COPD (11 (9) vs. 0 (0)) (*P* = 0.035) and dialysis (10 (8) vs. 0 (0)) (*P* = 0.048), implanted endovascular devices (42 (33) vs. 2 (5)) (*P* < 0.001) and artificial joints (11 (9) vs. 0 (0)) (*P* = 0.035) in the R group than in the control group. Regarding admission source, admission from the emergency department (ED) was more common in the control group (53 (41) vs. 39 (89)) (*P* < 0.001). In other words, patients without the risk of MDR bacterial infection were more likely to enter the ED. Overall, sepsis and septic shock occurred in 70% and 37% of patients, respectively.

**Table 1:** Clinicodemographic characteristics of the study population in each group at the time of blood culture

	All patients (n = 173)	R group (n = 129)	Control group (n = 44)	*P* value
Percentage of patients	100	74.6	25.4	
A: Resistant organism detected	15 (9)	15 (12)	0 (0)	
B: Antimicrobial use within 90 days	108 (62)	108 (84)	0 (0)	
C: Hospitalization within 90 days	87 (50)	87 (67)	0 (0)	
Age	73 (63–81)	75 (65–81)	72 (58–77)	0.042
Female sex	69 (40)	55 (43)	14 (32)	0.206
Body mass index	22 (19–26)	22 (19–25)	24 (21–26)	0.005
Coexisting condition				
COPD	11 (6)	11 (9)	0 (0)	0.035
Diabetes	67 (39)	46 (36)	21 (48)	0.156
Dialysis	10 (6)	10 (8)	0 (0)	0.048
Malignancy	36 (21)	30 (23)	6 (14)	0.175
Allergy to antibiotics	5 (3)	3 (2)	2 (5)	0.376
Implantable devices				
Endovascular device	44 (25)	42 (33)	2 (5)	< 0.001
Artificial joint	11 (6)	11 (9)	0 (0)	0.035
Admission source				< 0.001
Emergency department	92 (53)	53 (41)	39 (89)	
General ward	81 (46)	76 (58)	5 (11)	
SOFA score	8 (5–11)	8 (5–11)	7 (3–11)	0.529
Δ SOFA score	4 (1–8)	4 (0–8)	7 (3–11)	0.006
Lactate (mmol/L)	2.2 (1.3–3.9)	2.1 (1.3–3.5)	2.8 (1.3–4.9)	0.101
Sepsis	122 (70)	84 (65)	38 (86)	0.008
Septic shock	65 (37)	48 (37)	17 (38)	0.866

Values are presented as the medians (IQRs), n, or n (%)

*P* values were calculated with a two-tailed test

### Clinical characteristics and outcomes

We compared whether there were differences between the two groups in the clinical characteristics and prognoses of the patients after the BC test. The clinical characteristics of the study population in each group after the BC test are shown in Table 2. Empirical antimicrobials were administered in 81% of all cases. Bacteraemia occurred in 65 (38%) of all cases, of which 6 (3%) did not meet the criteria for sepsis. Of the nonseptic patients, 11.7% had bacteraemia. A review of medical records revealed that 50 (29%) of the total cases were not infectious, of which 29 (58%) received empiric antimicrobials. The de-escalation rate did not differ between the two groups. There was no difference in outcomes between the two groups regarding the number of days in the ICU and hospital, ventilator-free days, 28-day mortality, or discharge route. CDI and candidemia occurred only in the R group, although there was no significant difference in the incidence of either between the groups.

**Table 2:** Clinical characteristics after blood culture

	All patients (n = 173)	R group (n = 129)	Control group (n = 44)	*P* value
Bacteraemia	65 (38)	45 (35)	20 (45)	0.211
Septic shock	35 (20)	24 (18)	11 (25)	
Sepsis without shock	24 (13)	15 (11)	9 (20)	
No sepsis	6 (3)	6 (4)	0 (0)	
Infection site				
Respiratory tract	54 (31)	41 (32)	13 (30)	
Urinary tract	15 (9)	12 (9)	3 (7)	
Catheter-related bloodstream	7 (4)	7 (5)	0 (0)	
Surgical site	3 (2)	2 (2)	1 (2)	
*Clostridioides difficile*	0 (0)	0 (0)	0 (0)	
Abdomen	20 (12)	13 (10)	7 (16)	
Central nervous system	3 (2)	2 (2)	1 (2)	
Other	21 (12)	16 (12)	5 (11)	
None	50 (29)	36 (28)	14 (32)	
Empiric antimicrobial administration	141 (81)	108 (83)	33 (75)	0.198
De-escalation	59 (34)	44 (34)	15 (34)	0.998
Other treatment in ICU				
Intubation	45 (26)	33 (26)	12 (27)	0.825
Tracheostomy	47 (27)	37 (29)	10 (23)	0.443
Thoracic drainage	24 (14)	23 (18)	1 (2)	0.010
IPPV	125 (72)	88 (68)	37 (84)	0.042
NPPV	10 (6)	8 (6)	2 (5)	0.510
HFNC	28 (16)	20 (16)	8 (18)	0.677
CV	59 (34)	44 (34)	15 (34)	0.998
PICC	26 (15)	20 (16)	6 (14)	0.765
RRT	41 (24)	29 (22)	12 (27)	0.519
IABP	20 (12)	12 (9)	8 (18)	0.112
ECMO	11 (6)	9 (7)	2 (5)	0.437
TTM	13 (8)	7 (5)	6 (14)	0.078
Nutrition				
EN	76 (44)	57 (44)	19 (43)	0.908
TPN	18 (10)	17 (13)	1 (2)	0.030
Number of days in the ICU	10 (6–16)	11 (6–19)	10 (7–15)	0.239
Number of days in the hospital	48 (25–81)	49 (25–82)	43 (29–71)	0.475
Ventilator-free days	22 (5–27)	22 (1–28)	22 (11–26)	0.836
28-day mortality	31 (18)	23 (18)	8 (18)	0.958
Discharge route-no. (%)				0.556
Death	50 (29)	39 (30)	11 (25)	
Transferred to another hospital	71 (41)	54 (42)	17 (39)	
Discharged home	52 (30)	36 (28)	16 (36)	
HAI after ICU discharge				
CDI (test n = 37)	3 (2)	3 (2)	0 (0)	0.412
Candidemia	8 (5)	8 (6)	0 (0)	0.090

Values are presented as the medians (IQRs), n, or n (%)

### Organisms

The bacteria detected in the blood are shown in Tables 3. The most frequently detected organisms in the blood were *Escherichia coli* among gram-negative bacteria and *Staphylococcus aureus* among gram-positive bacteria. *Candida albicans* was the only fungus detected.

**Table 3:** Microbiologic results obtained through blood culture

	All (n = 68)	R group (n = 48)	Control group (n = 20)
Gram-negative bacilli			
*Escherichia coli* (non-ESBL)	10 (23)	6 (12)	4 (20)
*Escherichia coli* (ESBL)	6 (9)	6 (12)	
*Klebsiella pneumoniae* (non-ESBL)	5 (9)	2 (6)	3 (15)
*Klebsiella pneumoniae* (ESBL)	1 (1)	1 (2)	
*Klebsiella aerogenes*	1 (1)		1 (5)
*Enterobacter cloacae*	5 (7)	4 (8)	1 (5)
*Serratia marcescens*	1 (1)	1 (2)	
*Pseudomonas aeruginosa*	4 (6)	4 (8)	
Gram-positive cocci			
Methicillin-sensitive* Staphylococcus aureus*	9 (17)	5 (16)	4 (20)
Methicillin-resistant* Staphylococcus aureus*	3 (4)	3 (6)	
*Staphylococcus epidermidis*	3 (4)	3 (6)	
*Streptococcus pneumoniae*	1 (1)		1 (5)
*Streptococcus pyogenes*	4 (6)	1 (2)	3 (15)
*Streptococcus agalactiae*	1 (1)	1 (2)	
*Streptococcus* species	5 (7)	3 (6)	2 (10)
*Enterococcus faecalis*	2 (3)	2 (4)	
Anaerobes			
*Clostridium perfringens*	2 (3)	1 (2)	1 (5)
*Bacteroides fragilis*	1 (1)	1 (2)	
Fungi: *Candida albicans*	4 (6)	4 (8)	

Values are presented as the medians (IQRs), n, or n (%). *Streptococcus* species refers to *Streptococcus* species other than *Streptococcus pneumoniae* and *β-haemolytic streptococci*

The number of bacteria detected in the blood, particularly MDR bacteria, *Pseudomonas aeruginosa*, and fungi, is shown in Table 4. MDR bacteria detected in BCs were ESBL-producing *Escherichia coli*, ESBL-producing *Klebsiella pneumoniae*, and MRSA. These MDR bacteria were detected only in the R group (10 (8%) vs. 0 (0%)) (*P* = 0.048). All cases of *Pseudomonas aeruginosa* infection were also detected in the R group. However, no *Pseudomonas aeruginosa* resistant to three or more antimicrobial classes was detected. Thus, the number of cases of infections with MDR bacteria and *Pseudomonas aeruginosa* in the R and control groups was 14 (11%) and 0 (0%) (*P* = 0.014), respectively.

**Table 4:** MDR bacteria detected in blood cultures

	All patients (n = 173)	R group (n = 129)	Control group (n = 44)	*P* value
MDR bacteria	10 (6)	10 (8)	0 (0)	0.048
*Escherichia coli* (ESBL)	6 (3)	6 (5)	0 (0)	
*Klebsiella pneumoniae* (ESBL)	1 (1)	1 (1)	0 (0)	
*Staphylococcus aureus* (MRSA)	3 (2)	3 (2)	0 (0)	
Other	0 (0)	0 (0)	0 (0)	
MDR bacteria + *Pseudomonas aeruginosa*	14 (8)	14 (11)	0 (0)	0.014
Fungi: *Candida albicans*	4 (2)	4 (3)	0 (0)	0.305

Values are presented as n (%)

### Empiric antibiotics

Empiric antimicrobials administered after BC collection are shown in Table 5. Antimicrobials selected as empiric therapy did not differ between the two groups. The number of cases of appropriate antimicrobial spectrum coverage for the BC-positive cases in the R and control groups was 29 (64%) and 19 (95%) (*P* = 0.010), respectively. However, the 28-day mortality rate for BC-positive cases did not differ between the two groups.

**Table 5:** Empiric antibiotic use

	All patients (n = 173)	R group (n = 129)	Control group (n = 44)	*P* value
Antifungal	15 (9)	14 (11)	1 (2)	0.066
Anti-MRSA	42 (24)	30 (23)	12 (27)	0.592
Anti-*Pseudomonas*	77 (45)	61 (47)	16 (36)	0.208
Carbapenem	30 (17)	22 (17)	8 (18)	0.865
Anti-*Pseudomonas* penicillin	37 (21)	30 (23)	7 (16)	0.305
Anti-*Pseudomonas* cephem	8 (5)	7 (5)	1 (2)	0.351
Quinolone	2 (1)	2 (2)	0 (0)	0.555
Penicillin (excluding anti-*Pseudomonas*)	29 (17)	20 (16)	9 (20)	0.448
Cephem (excluding anti-*Pseudomonas*)	31 (18)	23 (18)	8 (18)	0.958
Macrolide	3 (2)	1 (1)	2 (5)	0.160
Sulfamethoxazole Trimethoprim	3 (2)	3 (2)	0 (0)	0.412
Metronidazole	4 (2)	4 (3)	0 (0)	0.305
Clindamycin	2 (1)	0 (0)	2 (5)	0.064
Antiviral	2 (1)	0 (0)	2 (5)	0.064
None	32 (18)	21 (16)	11 (25)	0.198
Bacteraemia	65 (38)	45 (35)	20 (45)	
Appropriate spectrum coverage	48 (74)	29 (64)	19 (95)	0.010
28-day mortality	15(23)	11 (24)	4 (20)	0.480

Values are presented as the medians (IQRs), n, or n (%)

## Discussion

We reported clinical and epidemiological data obtained from BC tests performed in an ICU in Japan. To establish guidance regarding the choice of antimicrobial therapy for ICU patients, we clarified the clinical characteristics, bacteriological test results, treatment, and prognosis of all patients with BCs collected in the ICU and examined whether the detection rate of MDR bacteria differed depending on the presence or absence of the risk of MDR bacterial infections. A total of 129 (74.6%) cases involved patients who exhibited at least one of our defined risks of infection with MDR bacteria, and 44 (25.4%) cases involved patients who did not; these patients were assigned to the R and control groups, respectively. Admission from the ED was more common in the control group (53 (41) vs. 39 (89)) (*P* < 0.001). Of the 173 cases with a BC, sepsis occurred in 70%, and septic shock occurred in 37%. Additionally, 50 (29%) of the total cases were consequently noninfectious, but 29 (58%) of the patients received empirical antimicrobials according to the medical records. This finding shows how difficult it is to determine the presence or absence of infection at the time of BC collection. The number of cases of infections with MDR bacteria and *Pseudomonas aeruginosa* in the R and control groups was 14 (11%) and 0 (0%) (*P* = 0.014), respectively. However, the antimicrobials selected as empiric therapy did not differ between the two groups.

This study was conducted in a Japanese mixed ICU and included all patients for whom BCs were performed for suspected new infections. The control group without the risk of MDR bacterial infections accounted for approximately 1/4 of the total. There were more admissions from the ED in the control group, reflecting the admission of new patients who were likely to have less exposure to antimicrobials. Sepsis cases were more common in the control group, which had higher ΔSOFA scores. In a situation in which sepsis is very common when infection is suspected, selecting a broad-spectrum antimicrobial agent in the ICU solely because of sepsis will result in a considerable increase in the use of broad-spectrum antimicrobial agents. Guidelines for sepsis do not recommend administration of broad-spectrum antimicrobials simply because of sepsis [10]. In fact, however, a cohort study of Japanese ICUs showed a trend towards the use of broader-spectrum antimicrobials for critically ill patients [20]. Therefore, to use antimicrobial agents appropriately to prevent the emergence of resistant bacteria, it is extremely important to cautiously assess the risk of infection with resistant bacteria without relying too much on the severity of the patient's illness. The number of cases of infection with MDR bacteria or *Pseudomonas aeruginosa*, identified by BC was significantly higher in the R group than in the control group (14 (11%) vs. 0 (0%)) (*P* = 0.014). However, the empiric antimicrobials used did not differ between the two groups. The appropriate antimicrobial spectrum coverage rate for BC-positive cases was lower in the R group. Therefore, broader-spectrum antimicrobials should have been selected for the R group at risk of MDR bacterial infections. In addition, the appropriate antimicrobial spectrum coverage rate for BC-positive cases was higher in the control group. Thus, we may have used too many broad-spectrum antimicrobials in the control group, which had patients without the risk of MDR bacterial infections. In the treatment of sepsis, the strategy of selecting broad-spectrum antimicrobials for empiric therapy and later reducing their use has been emphasized [10]. Broad-spectrum antimicrobial usage for more than 72 h was previously associated with the increased detection of new MDR bacteria [20]. However, in a multicentre study of a total of 152 ICUs in 28 countries, de-escalation of antimicrobial therapy within 72 h of empiric therapy was performed in only 16% of cases of severe infection [21]. Thus, the empirical use of broad-spectrum antimicrobials is likely to be prolonged, contributing to the emergence of MDR bacteria. Therefore, in cases in which MDR bacteria are unlikely to be the causative agent, the strategy of selecting narrow-spectrum antimicrobials as empiric therapy and switching to broad-spectrum antimicrobials as needed is important for preventing the spread of MDR bacteria. The targeting of all possible microorganisms should be attempted during the treatment of sepsis, but this strategy requires a thorough assessment of the risk of MDR bacterial infection at each facility. In this study, many patients without the risk of MDR bacterial infection were admitted to the ICU from the ED. However, the risk of MDR bacterial infection varies from case to case at each facility, and there are likely cases in which the risk of MDR bacterial infection is low. Careful selection of empirical antimicrobials by determining the risk of MDR bacterial infections in each case will prevent the spread of MDR bacteria. It is important to update epidemiological data daily with new cases and to take advantage of these data for subsequent cases. The three risk factors we chose for MDR bacterial infections were simple and reasonable because we did not detect a single case of MDR bacterial infection in the control group. However, risk factors for MDR bacterial infections should be determined with reference to clinical epidemiological data from each facility because the characteristics of the bacteria detected are different at each facility. Our study had several limitations. First, this was a single-centre case‒control study with a small number of cases, which means that its generalizability may be low, and it is critical to determine MDR bacterial infection risk based on local data from each facility. Second, this study did not consider cases of culture-negative sepsis. Even if sepsis is diagnosed clinically and the appropriate culture tests are performed, 30–60% of cases are culture negative [22]. Therefore, just because the patient was culture negative does not mean that the clinical symptoms were not caused by MDR bacteria. Third, fluid balance, vasopressor dosage and sedation level were not investigated. Thus, it is unclear whether the patients were truly in septic shock, and the number of septic shock cases may have therefore been overestimated. Finally, this study was based on the first BC test performed after ICU admission. Therefore, the findings may not be applicable to the results of secondary and subsequent BC tests. The risk of MDR infection at the time of a second or subsequent BC test will be considerably influenced by the extent of medical exposure following admission. Despite these limitations, our study provides a meaningful opportunity to promote antimicrobial stewardship in ICUs because few studies have clarified the clinical characteristics of patients with BC tests in the ICU.

## Conclusions

Even in critically ill patients in the ICU, MDR bacteria are unlikely to be detected in patients without the risk of MDR bacterial infections. Therefore, for such patients, a strategy of starting empiric narrow-spectrum antimicrobial therapy rather than empiric broad-spectrum therapy, carefully monitoring the patient, and adjusting the treatment as necessary should be considered. This strategy, in conjunction with daily updates of clinical and epidemiological data at each facility, will promote the appropriate use of antimicrobials and reduce the emergence of MDR bacteria in the ICU.

## Data Availability

The datasets used and analysed during the current study are available from the corresponding author upon reasonable request.

## Abbreviations

ICU: Intensive care unit
AMR: Antimicrobial resistance
MDR: Multidrug-resistant
BC: Blood culture
MRSA: Methicillin-resistant *Staphylococcus aureus*
HAI: Hospital-acquired infection
CDI: *Clostridioides difficile* Infection
ESBL: Extended-spectrum β-lactamase
BMI: Body mass index
COPD: Chronic obstructive pulmonary disease
SOFA: Sequential Organ Failure Assessment
VFD: Ventilator-free day
IPPV: Invasive positive pressure ventilation
NPPV: Noninvasive positive pressure ventilation
HFOT: High-flow oxygen therapy
CVL: Central venous line
PICC: Peripherally inserted central venous catheter
RRT: Renal replacement therapy
IABP: Intra-aortic balloon pumping
ECMO: Extracorporeal membrane oxygenation
EN: Enteral nutrition
TPN: Total parenteral nutrition
ED: Emergency department
IQR: Interquartile range
